# Natural Products and Cancer

**DOI:** 10.3390/nu15245064

**Published:** 2023-12-11

**Authors:** Yoichi Matsuo

**Affiliations:** Department of Gastroenterological Surgery, Nagoya City University Graduate School of Medical Sciences, Kawasumi 1, Mizuho-cho, Mizuho-ku, Nagoya 4678601, Japan; matsuo@med.nagoya-cu.ac.jp; Tel.: +81-52-853-8226; Fax: +81-52-842-3906

Natural and dietary compounds are known to offer protection and affect the pathogeneses of numerous chronic diseases. Recent evidence suggests that many chronic conditions, such as diabetes, cardiovascular disease, and cancer, are impacted by the consumption of fruits and vegetables. Several dietary compounds act as chemopreventive and chemotherapeutic agents against various forms of cancer [1]. The recent scientific literature suggests that the regular intake of food derived from natural products plays a critical role in the fight against cancer and other chronic diseases. Over the past few decades, several natural compounds have been discovered and are now widely used as anticancer agents, including paclitaxel, vinblastine, camptothecin, and oleuropein. In this Special Issue, Natural Products and Cancer, we highlight the anticancer activities of natural products and their underlying mechanisms, as demonstrated in various in vitro and in vivo models.

A total of 12 papers (6 original articles and 6 reviews) are published in this Special Issue.

Husam Qanash et al. reported on Saudi Sidr honey obtained from Ziziphus or the Lote tree. Saudi Sidr honey has potent antibacterial, anti-inflammatory, and antioxidant properties, making it effective in treating a range of health conditions, but few reports have described its effects on cancer. The present research was conducted to investigate the anticancer activity of Saudi Sidr honey against a variety of cancer cells, including colorectal cancer cells, breast cancer cells, and lung cancer cells. Husam Qanash et al. concluded that Saudi Sidr honey can inhibit cancer cell growth, causing apoptosis and arresting the cell cycle. Abuyaseer Abusaliya et al. reported on prunetrin (PUR), which is a flavonoid. Although some reports have suggested an anti-cancer effect of PUR on gastric cancer [2], no report has described its effects on hepatocellular carcinoma (HCC). This study revealed that PUR can potentially inhibit the growth of HCC by activating the caspase cascade and inhibiting the Akt/mTOR pathway. In addition to HCC, the effects of natural products on cholangiocarcinoma (CCA) have also been reported. Thanpisit Lomphithak et al. revealed that the target genes of flavonoids such as quercetin (QUE) and kaempferol (KEM) were enriched in G2/M-related genes, and a higher expression of G2/M signature genes was significantly associated with shorter survival in patients with CCA. Na-Ra Han et al. reported the effects of natural products on melanoma, the most invasive and lethal type of skin cancer. On the other hand, SH003, a mixture of natural products derived from *Astragalus membranaceus*, *Angelica gigas*, and *Trichosanthes kirilowii*, and formononetin (FMN), an active constituent of SH003, exhibit anti-cancer and anti-oxidant properties. In this report, Na-Ra Han et al. demonstrated the anti-melanoma effects of SH003 and FMN through the PD-1/PD-L1 pathway in melanoma cell lines, B16F10 cells, and CTLL-2 cells. Indeed, some reports have shown the effectiveness of immunotherapy for melanoma [3]. This paper shows that these natural products have the potential to be novel therapeutic agents for melanoma. Iera Hernandez-Unzueta et al. reported the effects of a natural product on prostate cancer. Prostate cancer is the second-most common cancer among men and the fifth leading cause of cancer death [4]. The most critical problem in the treatment of prostate cancer is that resistance to chemotherapy arises after first-line treatment. Thus, Iera Hernandez-Unzueta et al. focused on the cytotoxic capacity of the natural product by itself and when combined with existing anticancer drugs as an adjuvant agent. In this regard, the natural nutritional mixture, named Ocoxin, showed antitumor properties by itself against different primary and metastatic cancers, increasing apoptosis and causing cell cycle arrest [5]. The authors revealed that the combination of an Ocoxin supplement with chemotherapy showed a higher cytotoxic effect than chemotherapy alone and reversed the chemoresistance conferred by cancer-associated fibroblasts (CAFs) and osteoblasts in both in vitro and in vivo models. Therefore, they concluded that Ocoxin is a good candidate for further study in combination with current treatments for patients with prostate cancer. As mentioned in that paper, we have begun to see papers reporting the effectiveness of combination therapy with existing anticancer drugs and natural products. In this Special Issue, Jankiben R. Patel et al. described the effects of combination treatment with natural products, glyceollins, and existing chemotherapy for breast cancer. Glyceollins are members of the flavonoid family of soy-derived phytochemicals, and previous reports have demonstrated the effect of glyceollins on cancer prevention [6]. In this report, the authors demonstrated that, while glyceollin + lapatinib treatment had comparable inhibitory effects on proliferation and migration in breast cancer cell lines, combination treatment selectively induced the S and G2/M phase cell cycle arrest of LTLT-Ca cells, which was mediated by decreased cyclin B1. As described above, we are looking forward to determining the effects of combination therapy with natural products and anticancer drugs.

With regard to the six reviews published in this Special Issue, Pratibha Pandey et al. focused on exploring the potential of melittin, a peptide component of bee venom that has shown promising potential in the treatment of several human cancers, including breast, stomach, lung, prostate, ovary, kidney, colon, gastric, esophageal, and cervical cancers, as well as melanoma, osteosarcoma, and hepatocellular carcinoma. The authors demonstrated the effectiveness of melittin in producing apoptosis, necrosis, mitochondrial disruption, and cell cycle arrest, in preventing angiogenesis, and in suppressing cancer cell invasion and metastasis. Two reviews on flavonoids are published in this Special Issue. The antitumor effects of flavonoids have been known for a long time. First, Dominika Wendlocha et al. reviewed the effects of flavonoids on breast and gynecological cancer. In that report, compounds such as KEM, myricetin (MYR), QUE, fisetin (FIS), galangin (GAL), isorhamnetin (IZO), and morin demonstrated positive results in preclinical studies. Second, Shu Chyi Wong et al. reviewed the anticancer mechanism of flavonoids on high-grade adult-type diffuse gliomas. That summary of the effects of flavonoids on gliomas is an extremely valuable report. Huang Q et al. also reviewed the effect of natural products on gliomas from the standpoint of immunotherapy. They proposed that the use of natural products has emerged as a promising and safe strategy for glioma therapy, since most possess excellent antitumor effects and immunoregulatory properties by reversing the glioma immunosuppressive microenvironment (GIME). These mechanisms have not yet been fully elucidated, and future research is expected. Taghreed A. Majrashi et al. reviewed phytochemicals, such as apigenin, baicalin, and curcumin, as therapeutic agents. Kok-Lun Pang summarized the anticancer effects and biological activities of tocotrienol, a type of vitamin E, as contained in a database. Tocotrienol is well known for its anti-cancer and other biological activities.

The articles in this Special Issue show that compounds derived from natural products have anticancer effects. In the future, these results will be applied clinically. I hope that this will be of benefit to many patients.
